# Foreign Medico-Psychological Literature

**Published:** 1863-01

**Authors:** 


### FOREIGN MEDICO-PSYCHOLOGICAL LITERATURE.

Our Retrospect of current Foreign Medico-Psychological Literature embraces the following subjects:

1. The Criminal Responsibility of Deaf Mutes.
2. The Mutual Life of Man in its Organical Development, Rejuvenescence, and Health.
3. Account of an Epidemic of Hysterical Demonomania.
4. Dr. Parigot on Gheel.

### 1.—*The Criminal Responsibility of Deaf Mutes.*

The following is a report of a decision by Dr. Behr, of Bernburg, in the case of a deaf mute accused of premeditated incendiarism:—

The facts communicated by the Official Reports are, that W. Franke, 24½ years old, was born deaf and dumb, and from his eighth to his fourteenth year was instructed in the Deaf and Dumb Institution, at Halle. He learnt reading and writing tolerably well, received religious instruction, and was admitted to the Lord's Supper, in the Market Church, by the chief minister. After his return to his father's house, in Harzgerode, and till the time of the incendiarism, Franke was employed as a wagoner, and this avocation greatly developed his bodily strength. According to the deposition of witnesses, and still more according to Franke's own declaration, he often received severe blows from his father while hard at work, and in the opinion of the son, his two healthy sisters were always preferred by his father. On the morning of the 12th May, 1858, the father fell into a violent dispute with his son at breakfast, in which he (the son) gave his father a box on the ear, pushed about his eldest sister, who was in the room, and became altogether very much excited. Whilst the father was in the bedroom, the son, in the presence of his sister, took some matches from the cupboard, put them into his waistcoat pocket and went to the farm-yard. A short time after he returned again to the room, and his sister now observed that fire had broken out from the barn; the fire, however, was subsequently extinguished. During the fire the culprit entered the house, clapping his hands, and repeating often, "Good—good." On his arrestment, immediately afterwards, he confessed directly, as well by gestures as by written answers to written questions, that he had set fire to the hay and barn by means of the matches.

The examination into the bodily and mental condition of the prisoner was made with praiseworthy care by the district physician, and impressed him with these convictions: that Franke was possessed of a healthy although somewhat tardy mental organisation; that he was in a condition to distinguish right from wrong, good from evil, &c.; that he could judge of lawful and unlawful actions; that he was conscious, before and after the act of incendiarism, of its culpability, and that he acted with deliberation and freedom in the performance of the deed. The district-physician declared, therefore, that Franke was in a condition to be charged with the responsibility of his actions.

The following circumstances, however, appeared to the district-physician to demand consideration: to wit, that Franke was deaf and dumb, and that consequently he could not possess any idea of the nature or the consequences of his act, because he had never seen a great fire, and therefore he could not be pronounced fully responsible. These opinions were maintained by the district physician all along. The inconsistency in the opinion—that Franke, in spite of his cognate deaf-mutism should be judged responsible, but that on account of his want of knowledge of a fire and its consequences, and because of his peculiarity as a deaf and dumb person, the full responsibility could not exist—induced the Ducal Government to submit the case to Dr. Behr, who gave the following opinion upon it:—

Among the grounds upon which punishment is remitted or mitigated, the condition of deaf-mutism is not mentioned in the penal code of the Duchy of Anhalt-Bemburg, neither does the German criminal law contain a legal definition of the responsibility of the deaf and dumb. The question whether, and in how far, the action could lawfully be imputed to Franke must therefore be left to the judge, and the opinion of this court upon the responsibility of Franke can only be formed upon medical-technical grounds.

According to Casper (*Manual of Judicial Medical Biology*, 1858) the extent of responsibility depends upon immutable psychological, natural laws, the knowledge of which exists in the consciousness of every man. The knowledge of good and evil which naturally exists in every man becomes discernment, and the perfect freedom of the healthy man to allow his actions to be guided by the good or evil tendency is called free will. Every man must, in spite of his freedom of choice, endeavour by his actions to withstand the enticements of the evil tendency; if he does not do so rebuke is conveyed by his internal judge, conscience.

Every man living in society, who has attained to the normal development of his mental faculties, has experienced and knows that society does not and cannot rest content with this monitor, and that, corresponding to the demands of natural justice, punishments have been established for actions opposed to the moral law. According to this the actions of a man are measured and estimated; and so long as he is in undisturbed possession of his mental faculties he is held responsible for the good and evil consequences of his actions.

The illegal action of Franke does not stand isolated, but the incitement to it arose gradually from discontent consequent upon frequent and often violent quarrels with his father, and served to appease his anger, which had been excited by the previous dispute. The excitement did not prevent his acting deliberately, but was yet so powerful that after doing the deed Franke rejoiced aloud at it, and instead of concealing the action, he immediately confessed himself the author of it. He felt repentance for the first time when the consequences which his act might have occasioned were explained to him.

The brief extracts from the reports concerning the incendiarism of

Franke would have proved his perfect responsibility, before, during, and after the action, by universal testimony, if the limited amount of mental power which he, as a man born deaf and dumb had attained, had not to be taken into consideration. The greater number of deaf and dumb persons are originally endowed with all the mental faculties, and are therefore not only capable of performing simple mechanical occupations, and becoming self-supporting and useful members of the community, but individual talent is even found amongst them; and they may become artists by the preponderance of one individual endowment. Such cases are, of course, seldom to be met with. Generally, however, the mental faculties are not developed, and remain stationary at the lowest point, because the animating mental intercourse with contemporaries, enjoyed by the simplest peasant, is cut off from the deaf and dumb, or enjoyed in the lowest degree. All laws, all authors, attribute, therefore, value to the instruction which the deaf and dumb have enjoyed, and it cannot be contradicted that a special tuition works as a blessing, if it can only so far impart to the deaf and dumb some dexterity in elementary knowledge, and some understanding in religious and moral things. How much or how little, however, even the best school, even the best teacher, can accomplish by the improvement of the faculties of these unfortunates, to which the natural helplessness of the deaf and dumb raises an unconquerable barrier, Casper (*Vergl. S.* 733) we have, alas! had opportunity to experience, to the fullest extent, by the continually occurring examinations into the state of mind of the deaf and dumb. Even if it be possible in the case of the well instructed deaf and dumb, to investigate the degree of their improvement and their spontaneous power, so that, at least according to Siebenhaar (*Encyclopædic Manual of Judicial Medicine, &c.*) it is not permitted to be neglected in judicial medical opinions, still the same absolute certainty concerning their real mental condition can never, with the greatest care, be attained as with those who hear, and the character of the whole psychical life of the Deaf and Dumb furnishes a very important reason, for, in general, mitigating judgment upon their illegal actions.

Let us apply these experiences to the case in question, and we find that Franke, when a boy of fourteen years of age, returned home from the deaf and dumb institution at Halle, and was immediately made use of in agriculture, and later in carrier work, corporeally perhaps, continuing normally to improve, but not, however, similarly improving in his mental condition; so that we must admit him to be, in a mental point of view, a boy of fourteen, who had not added to what he had acquired in Halle. We find proofs of these opinions in his answers to the written questions; the simplest of which he not unfrequently misunderstood. Thus he answered to the question: "Who lighted the fire in the barn? by "No" and the same to the question: "For what did you use the matches?" &c. Very many of his answers, even to questions the sense of which he (Franke) understood, proved his low intelligence, and produced the impression that he had not, for one of his age, attained the normal development of the mental faculties; indeed, that his answers must proceed from a child very backward mentally, and of from ten to fourteen years of age. Franke knew, as every minor not quite an imbecile, of from twelve to sixteen years of age knows, to distinguish good from evil; knew that fire-raising was punished; he named even the term "Penal Code," without however associating any idea with it; and because his father treated and clothed him badly and gave him no money, he wished to frighten and move him to be less severe: and thinking that the fire lit by him would not spread further, he set fire to the barn, but wished to go no further. That Franke had no idea of a fire and its consequences, and that he was not even acquainted with fire as a means of destruction (Declaration of the District Physician in the Certificate), does not appear in the evidence. On the contrary, it is perfectly admitted, that in his business as a wagoner and also in his domestic occupations, he must have become acquainted with fire as a means of destruction. Relying upon this knowledge, he took the matches, in order, as he himself stated, to burn the barn over the stable. It must also be observed that Franke had seen the power of fire as a means of destruction when he saw a burning wood at Harzgerode. (*Vergl. Gerichts-Acten.*)

The final result of these inquiries is: that, Wihl Franke of Harzgerode, twenty-four and a half years of age, deaf and dumb, at the time of the perpetration of the act of incendiarism, was perfectly physically developed; but being deaf and dumb, had not attained the full development of his mental faculties; consequently he was in the condition of a minor of from fourteen to sixteen years of age, and only partially responsible. (*Casper's Viertljahrsschrift, July,* 1862.)

---

2.—*Schultz and Hegel.—The Mutual Life of Man in its Organical Development, Rejuvenescence, and Health. By* FERDINAND SCHNELL.

OUR author desires to render the results of the researches of Schultz Schultzenstein, contained in his comprehensive work entitled *New System of Psychology,* (Berlin, 1855,) accessible to the cultivated reader. As an attempt at forming a Psychology from the Hegel point of view, the new system, and consequently our abstract of it, deserves attention. It is our purpose, not so much to give an epitome of Schnell's abstract, but rather to discuss the grounds upon which the system is based.

According to Schultz Schultzenstein, the body is merely the visible form of the mind; both body and mind are subject to the same laws of life; the life of both expresses itself in the same principal functions, the assimilation and the formation of material. Whilst the mental principle forms a spiritual body, it developes itself organically, from a germ, like the natural body, and in connexion with and by means of the same. The material which the mind assimilates and from which it forms itself is the world. The mind must digest the world, transform it into its flesh and blood. The comparison between the growth of the body and that of the mind is carried out in this book into the minutest details, and often in a playful manner. Thought is the stomach of the mind, learning is mental eating and digestion; schools are mental eating houses; teachers mental cooks; the insane are defined as people whose mental stomachs, whose organs of secretion, have become deranged, &c. The outer world affords, however, only the excitement to and the material for the formation of the mind; this formation itself is the product of the creative power of the mind itself, "it is only rendered fruitful through the light and life of the world of nature and of man." The development of the mind proceeds gradually, step by step, and is subject to the law of rejuvenescence or the second birth. With the discovery of this law by Schultz, (so, at least, thinks Herr Schnell) a new era arrives for Church, State, and School, which must transform all science to which, hitherto, "secure basis has been wanting." Law-givers and judges, theologians and physicians, educators and teachers, find here the foundation for a true scientific creed.

Certainly, Christianity has already proclaimed the law of rejuvenescence, or the second birth, but without understanding it. Wherein, then, consists this law? Our author answers here, as in the whole book, not in plain language, but figuratively, apparently under the impression that such a form of expression is more easily understood. We will now allow him to speak for himself.

"As the plants sleep in winter, but in spring the buds unfold and the leaves and shoots come forth to the day, as they experience in this way a new birth, year by year, whilst they gradually advance to a higher form; in like manner does the mind develope itself. Its life also proceeds by a continual change, a decay and removal of all its forms. The feelings, instincts, imaginations, acts of free will, expire at certain periods, like the leaves of the plant, and are then formed anew. They decay, in order to come forth with fresh vigour and strength. The whole fable of the phœnix, who, from century to century, arose more beautiful from its ashes, is here an actual fact. The mind retires, from time to time, into itself; at each new act of formation the former material of development is refined, and the old form is rejected as a 'cast off skin.' Everything that is commonly understood by passion, in the worst sense of the word, vices, errors, &c., is compared to matter which, resisting the powers of secretion, must be thrown off by the system. Contrasting feelings, antagonistic ideas and expressions of the will, rest upon the opposition between formation and decay, and are necessary to the life of the mind. So past joy becomes grief, out of which, by means of the second birth, a higher joy is developed. 'To forget' is the completion of the act of moulting."

If the author of our book supposes that Schultz Schultzenstein has discovered something new in propounding this law, it may be as well to remind him that this is only an idea of Hegel's, applied to psychology: that the mind may be no finished, consistent being, but a living, never resting process of development, in antagonism and progress of the same into unity. The abstract idea has here only found a concrete, figurative representation. That this is the case will be seen by a short review of the system. Out of the mind, as the embryo of the soul, proceed feelings and impulses, and gradually their affections and passions, and in these the life of the soul evolves itself into a multiplicity. The most comprehensive of all feelings is the universal feeling of truth, which the system calls faith. This process forms the first and second step of mental life. At the third step the soul transforms the feelings into living ideas, and impulses take the form of acts of the will. Multiplicity unites itself here to the unity of self-consciousness. Upon this third step, as it is impressively remarked, the life of the soul does not remain inchoate and in scattered elements, but fits itself into the organic unity, into the self-consciousness of individuality and its freedom together. If the mental organism has reacted, a perfect living organism of ideas results, of which free will is the expression; and then the fourth and last step is attained, namely, the living reason. Here the multiplicity of ideas is bound up with the perfect unity of the living kingdom of thought, into which the individual mind ascends. Who does not find in this unfolding and concentrating the psychological fundamental views of Hegel? According to him consciousness developes itself in three stages in the mind of man. It appears firstly and directly in the form of intuition, and therewith art; these pass over into the form of representations or religion, and these last afterwards raise themselves to the form of ideas, and therewith philosophy. These principal features of Hegel's Psychology meet us everywhere in this system. Here also all natural imaginations commence with the intuition of thought. The clearness of thoughts awakens the slumbering strength of the soul, and these are worked up by it, first into thought-pictures, that is to say, organic products in the instruments of thought, and then into feelings. The highest significance is ascribed to the symbol which nature offers and art provides, because in it alone the soul comprehends, at this stage of its development, higher ideas. Feelings are dark representations, the most comprehensive of which is faith, by Hegel designated "religion," but the matter is the same. Whilst, in conclusion, the habitual representations, *i.e.*, the "feelings," are transformed into mental representations, *i.e.*, "ideas," through the process of thinking, and the condensed unity of these ideas is exhibited in this philosophy as the great aim of all human development; the third and fourth stages of our system are included in the third period, according to Hegel. It is evident, then, Schultz Schultzenstein has discovered nothing really new. The praise awarded to him by the author of our book, belongs, in reality, to the Master Hegel, whose dialectic penetration is sorely missed in this work. We repeat our introductory remark, that Hegel's philosophical hypotheses and method have been applied to the domain of Psychology, and it is in the nature of things, that exactly here they should cast their deepest shadow, since intellectual remarks are not sufficient for so obscure a field, and certainly no reliable observations can be obtained through the aid of an artificial system.

### 3.—*Account of an Epidemic of Hysterical Demonomania.* By Dr. Constans.

In the ancient province of Chablais, now in the arrondissement of Thonon (Haute-Savoie), is a commune of 2000 inhabitants named Morzines, situated at the extremity of the valley of Aulph. It is separated from Valais by a mountain only; its altitude is about 1500 metres, the climate severe, and the vegetation tardy. The people are very poor, and, occupying the basement story of wretched huts, they live in a half-asphyxiated state, huddled near cast iron stoves heated even to redness. Their food consists of barley bread, potatoes, and smoked meats; and for drink, water, always very cold, is alone used. Their aspect, as a rule, is pitiful, the lymphatico-nervous temperament predominates, childhood is sickly and not easily passed, the fecundity of families is very great, adult age is prematurely decrepit, and old age rare. In other respects, these people are gentle, honest, obstinate, very devout, and excessively superstitious; little intelligent at the best, their judgment is further obscured by a multitude of absurd beliefs.

In March, 1857, two little girls, blondes, very pious, puny, but withal healthy, became the subjects of certain extraordinary attacks. Presently the affection extended to others, and in seven months twenty-seven persons, children, young girls, and women, were seized by it. It was averred that during the paroxysms of the affection, the children spoke French with surprising facility, or responded in German or Latin, lost all family affection, became surprisingly insolent and impious, and exhibited extraordinary physical powers, four men being insufficient to restrain a single child. Moreover, the children would climb in the twinkling of an eye to the tops of trees, and then turn somersaults, or leap from one tree to another, removed many metres, and descend to the ground head downwards.

Towards the end of 1860, the number of "possessed" (for as such they were regarded in the commune) had increased to 110; and in April, 1861, the Minister of the Interior directed Dr. Constans, Inspector-general of Lunatics, to visit Morzines, and make inquiry into the nature and causes of the outbreak, and to take such steps as might be needed to put an end to it. When Dr. Constans reached Morzines he found the population much depressed, every one going in fear of devils, and a deep irritation reigned against the *sorcerers, the authors of the evil.* The treatment of the affected up to this period had consisted in parental intimidation, exorcisms, pilgrimages, and magnetism. Seventeen of the twenty-seven persons attacked in the first seven months of the epidemic had been cured, it was stated, by exorcism. Certain children had recovered spontaneously, and in others the attacks had yielded to promises, or threats of death.

Dr. Constans examined sixty-four of the "possessed." They were mostly celibates, hysterical, chloro-anæmic or scrofulous, and suffering from gastralgia, amenorrhœa, or dysmenorrhœa; the appetite was capricious, the sleep inconstant and light. Idle, loquacious, exalted, and fantastic, they flocked together, card-playing, exciting themselves, mutually and masking the insufficiency of their aliment by the immoderate use of black coffee. Everything gave occasion for a paroxysm, but nothing produced one so surely as the expression of a doubt that they were *possessed.* The paroxysm was ushered in by yawnings, pendiculations, startings, choreiform jerks, alternations of dilatation and contraction of the pupil, and a frightened aspect. Cries, vociferations, and oaths supervened. The physiognomy became dejected and assumed an expression of frenzy. The respiration was panting, and the movements, at first confined to the superior parts, extended successively to the trunk and extremities. Aggression commenced; furniture, chairs, or stools were cast at the spectators; then the convulsionnaires precipitated themselves upon their parents or upon strangers, struck them and struck themselves, bruising the chest or body, whirled about now in one direction, then another, and cast themselves on the back, starting up again as if it were on the rebound of a spring. No erotism mingled with the idea of demoniacal possession, and the affected never uttered obscene words or were guilty of lubrical actions. In the most disordered actions they never exposed the person. The paroxysm endured from twenty to twenty-five minutes, the pulse becoming enfeebled or lost, but the beating of the heart remained normal, while the hands were icy and the feet cold. Towards the decline of the paroxysm, the noise became less, the movements diminished in rapidity, there were eructations, the affected looked around with astonishment, arranged their hair, replaced their caps, drank several mouthfuls of water, and recommenced work, declaring that they felt no lassitude, and remembered nothing. It was evident, however, that the first assertion was not altogether true, and they heard and saw perfectly during the attacks; closing the eyes if menaced with a blow in the face, and avoiding, under all circumstances, places or bodies which might injure them when they cast themselves upon the ground.

Dr. Constans looks upon the outbreak as of an hysterical character, and that the affected were not altogether responsible for their acts. To this conclusion Dr. Legrand du Saulle (from whose abstract of Dr. Constans' account we derive the foregoing facts) demurs. Milder means proving unavailing, Dr. Constans quickly brought the epidemic to an end by having the priest of Morzines removed, and requiring the commune to be occupied by a brigade of gendarmerie and a detachment of infantry. The people were intimidated, and the "possessions" ceased. (*Ann. Médico-Psychologiques*, October, 1862.)

---

### 4.—*Dr. Parigot on Gheel.*

WE regard Dr. Parigot with such sincere friendship and respect as an upright man, as a philanthropist, and as one who, in advocating enthusiastically the perfection of a particular system of managing the chronic insane, has directed attention to what is admirable and useful in that system, that we deeply regret having inadvertently and unconsciously given him occasion for complaint. Our offences are enumerated in a letter published, p. 237, No. 2, *American Journal of Insanity*, October, 1862. The first of these is, that we have published a confidential letter "which he never thought of making public." It is perfectly true that a communication addressed to a correspondent did appear in our pages. It is further true that every word of a private or confidential character—and they were many—was carefully deleted; that our contributor has communicated to the writer the grounds upon which the purely professional paragraphs were introduced into the Article, "Colonization of Lunatics by the Legislature;" and, lastly, that these grounds consisted in a desire to do honour to a distinguished collaborateur, to announce to his friends in Europe what was conceived an improvement and development in his tone of thinking, if not in his opinions, and to record a most valuable contribution to the history of Gheel. The second offence attributed to us is, that we accused Dr. Parigot of proposing that asylums should bear upon their portals, *Lasciate ogne speranza voi ch'entrate.* We do not pretend to know the precise meaning which our friend attached to such expressions as, when describing superintendents of asylums, p. 280, vol. xix. of this Journal, he calls them "speculators and traffickers in madness," as those "who, on favourable occasions, only seek to retain captive unfortunate beings whom, most frequently, unnatural relations, from disgraceful motives, wish to get rid of," &c. But the impression was, and is, that the description must apply to establishments upon which such an inscription, suggested by himself, would have been most appropriate, but which are, happily, not now applicable to the asylums in this country. The third offence of which we are accused is in representing Dr. Parigot's views conveyed in the excellent letter addressed to our correspondent as "the legacy of one dead to European civilization," in other words, the deliverance of one alive to American civilization, and who had repudiated and denounced the modes of managing and treating the insane which still linger among us as antiquated, delusive, unscientific, and commercial; remarks which Dr. Willers Jessen, p. 612, vol. xiii. of this Journal, stigmatizes as "spiteful," but which we refrain from characterizing at all.

The fourth and final count of the indictment against us is, or appears to be, that we differ from him in opinion upon a great number of most important topics; and to this, and to this alone, but with unfeigned and undiminished esteem for our antagonist, we plead guilty.

